# Ultrasound-Guided Pulsed Radiofrequency of the Sciatic Nerve for Chronic Lower Limb Pain: A Real-World Cohort Experience

**DOI:** 10.7759/cureus.109155

**Published:** 2026-05-18

**Authors:** Eduardo González Godoy, Patricio Cardoso, Iris V De la Rocha Vedia, Ángel Plasencia Ezaine, Rocío Arenal López, María L Méndez Leo, Aitor Rojas Sánchez, María D Ruiz de Castañeda Zamora, Maruja Fernández Ordóñez, Marco Ramírez Huaranga

**Affiliations:** 1 Chronic Pain Unit, Hospital General Universitario de Ciudad Real, Ciudad Real, ESP

**Keywords:** chronic pain management, lower limb pain, opioid-free analgesia, pulsed radiofrequency treatment, ultrasound-guided

## Abstract

Background: Chronic neuropathic or mixed lower limb pain represents a frequent challenge in pain management units. The sciatic nerve ultrasound-guided pulsed radiofrequency (PRF) is a minimally invasive neuromodulation technique that may provide analgesia without thermal nerve injury.

Objective: To describe the outcomes of ultrasound-guided pulsed radiofrequency of the sciatic nerve in patients with chronic lower limb pain.

Methods: A single-center, retrospective, observational study including patients treated with ultrasound-guided sciatic nerve PRF between January 2020 and November 2025. Collected variables included clinical information, visual analogue scale (VAS), EuroQol-5D, previous analgesic consumption, complications, and global subjective perceived improvement. Pre- and post-treatment outcomes were compared using paired Student’s t-tests.

Results: A total of 266 patients were analyzed (152 (57.1%) women; mean age 67.8 ± 13.1 years). Overall clinical improvement (VAS reduction ≥3 points) was achieved in 181 (68.05%) patients, and 61 (33.83%) of them reduced analgesic consumption. The highest response percentages were observed in neurogenic claudication (83.3%), ankle/foot pain (80.8%), distal neuropathic pain (80%), and ischemic pain (71.4%). Significant improvements were observed in VAS and EuroQol-5D scores (p < 0.0001). Mean duration of clinical benefit was 5.1 ± 1.4 months, with effects persisting for up to 10 months in some patients. Only mild, transient complications were reported in 11 cases (4.14%).

Conclusions: Ultrasound-guided pulsed radiofrequency of the sciatic nerve appears to be a safe, minimally invasive, and potentially effective option for chronic nociceptive, neuropathic, or mixed lower limb pain. This study is conceived as one of the largest clinical published investigations, contributing to real evidence on patients and highlighting the clear necessity of additional controlled studies.

## Introduction

Neuropathic or mixed pain in the lower limbs secondary to radicular pain, neurogenic or vascular claudication, metabolic, traumatic, or post-surgical origin, among others, represents a frequent therapeutic challenge in pain units. In this context, pulsed radiofrequency (PRF) is a minimally invasive therapeutic alternative with a good safety profile for modulating pain conduction [1,2].

PRF is used on motor nerve structures, such as spinal roots or the sciatic nerve, because its short, repetitive pulses with interspersed pauses allow heat dissipation, generally maintaining temperatures below 42°C and thus avoiding thermal injury [3,4]. This ability to neuromodulate without destruction makes it an attractive alternative when dealing with mixed peripheral nerves where maintaining nerve structural integrity is required [5,6].

The sciatic nerve constitutes the main neural pathway supplying sensitive and motor innervation to the lower limbs, including the thigh, knee, leg, ankle, and foot. From an anatomical and neurophysiological perspective, it represents a logical therapeutic target for neuromodulation in a wide range of lower limb pain syndromes [7,8]. Furthermore, PRF induces changes at the level of neuronal signaling, glial activity, synapses, inflammatory regulation, and even modulation of ion channels and neurotransmitters, which supports its use as a pain neuromodulation technique [2,3].

Therefore, sciatic nerve pulsed radiofrequency (PRF) could be a therapeutic alternative by modulating the transmission of nociceptive, somatic, and neuropathic pain impulses in the lower extremities (neuropathies, radicular pain, distal pain from peripheral injury, postoperative pain, phantom limb pain, referred joint pain, neurogenic claudication, among others) [9-13].

The objective of this study is to describe our clinical experience using ultrasound-guided PRF of the sciatic nerve for the treatment of chronic lower limb pain, evaluating not only its effectiveness, safety profile, and complications, but also its impact on pain intensity, quality of life, and analgesic consumption across different diagnostic subgroups.

## Materials and methods

This was a single-center, observational, retrospective, descriptive, and analytical study in patients treated by ultrasound-guided sciatic nerve PRF for the treatment of chronic pain in the lower limbs during the period January 2020 to November 2025 in a Spanish third-level hospital.

The inclusion criteria included patients with lower limb pain refractory to optimized analgesic treatment and hygienic-dietary measures, with signed informed consent. Exclusion criteria included repeated PRF procedures back in time, incomplete clinical records regarding the main study variables (Visual Analogue Scale or VAS [14], EuroQol-5D [15], analgesic treatment, etc.), and the performance of additional interventional pain procedures during the follow-up period.

Sciatic nerve PRF was performed according to the standard protocol of the Pain Unit, by eight physicians. 

The procedure was performed in the ultrasound-guided technique room with the patient in the prone position, using standard monitoring and aseptic conditions. There were no special patient preparation needs.

**Figure 1:**
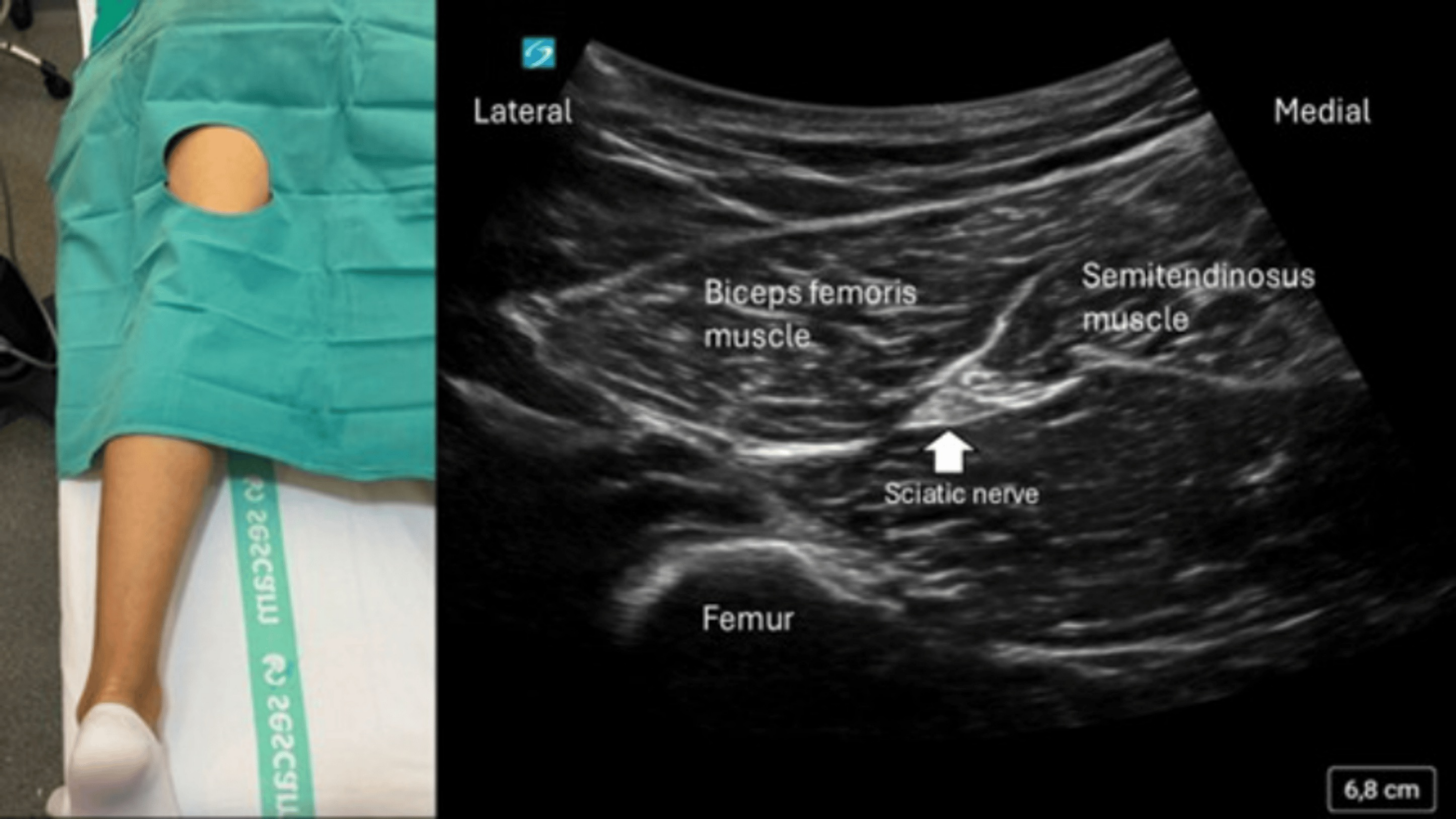
Sciatic nerve identification in the subgluteal approach (patient position).

An ultrasound machine and a convex probe were used to identify the sciatic nerve in the gluteal, subgluteal, or proximal thigh region (Figure 1). The approach was chosen based on where we found the best image. 

A radiofrequency device and a 22-G radiofrequency needle with a 10 mm active tip were used.

**Figure 2:**
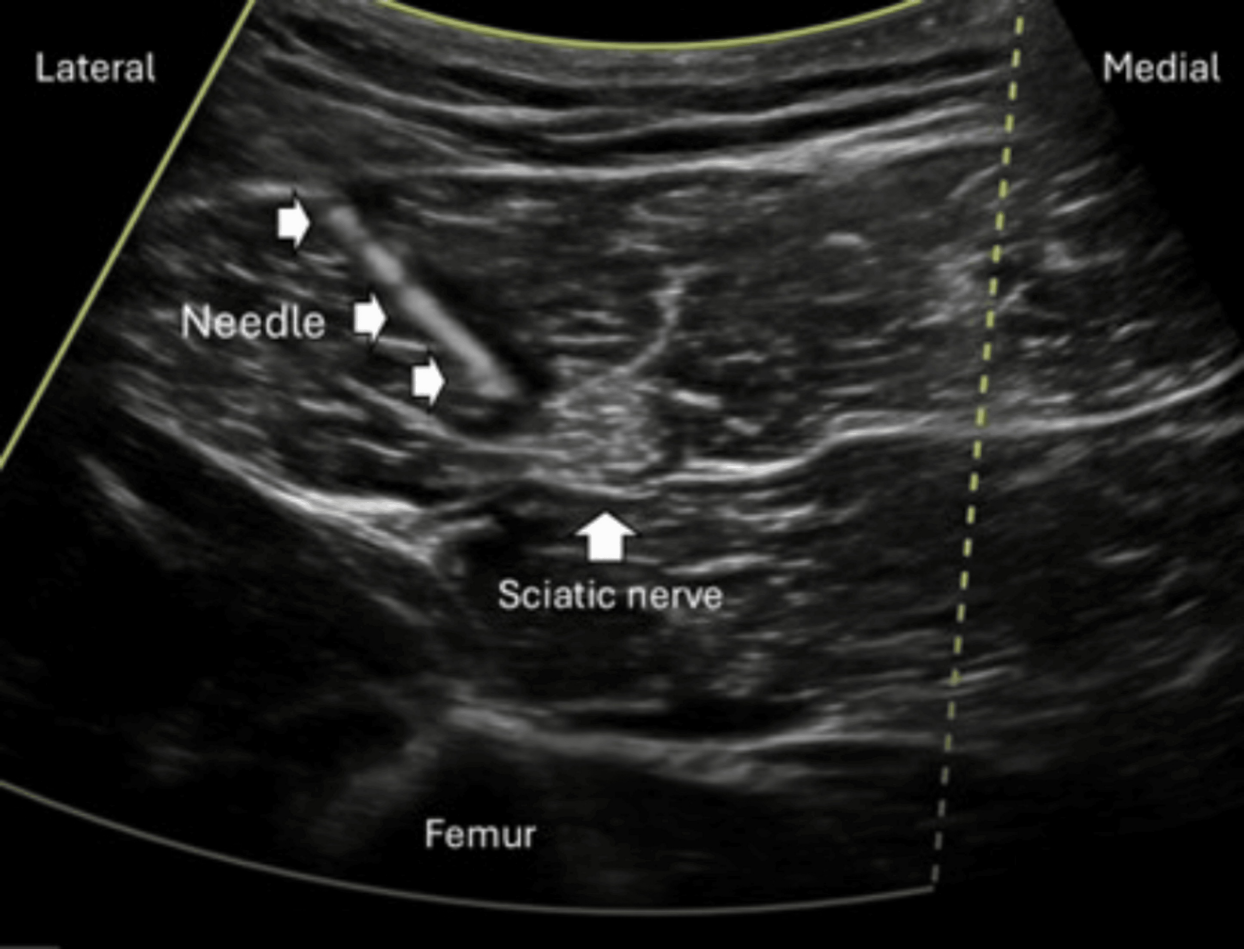
Stimulation of the sciatic nerve with the radiofrequency needle.

**Figure 3:**
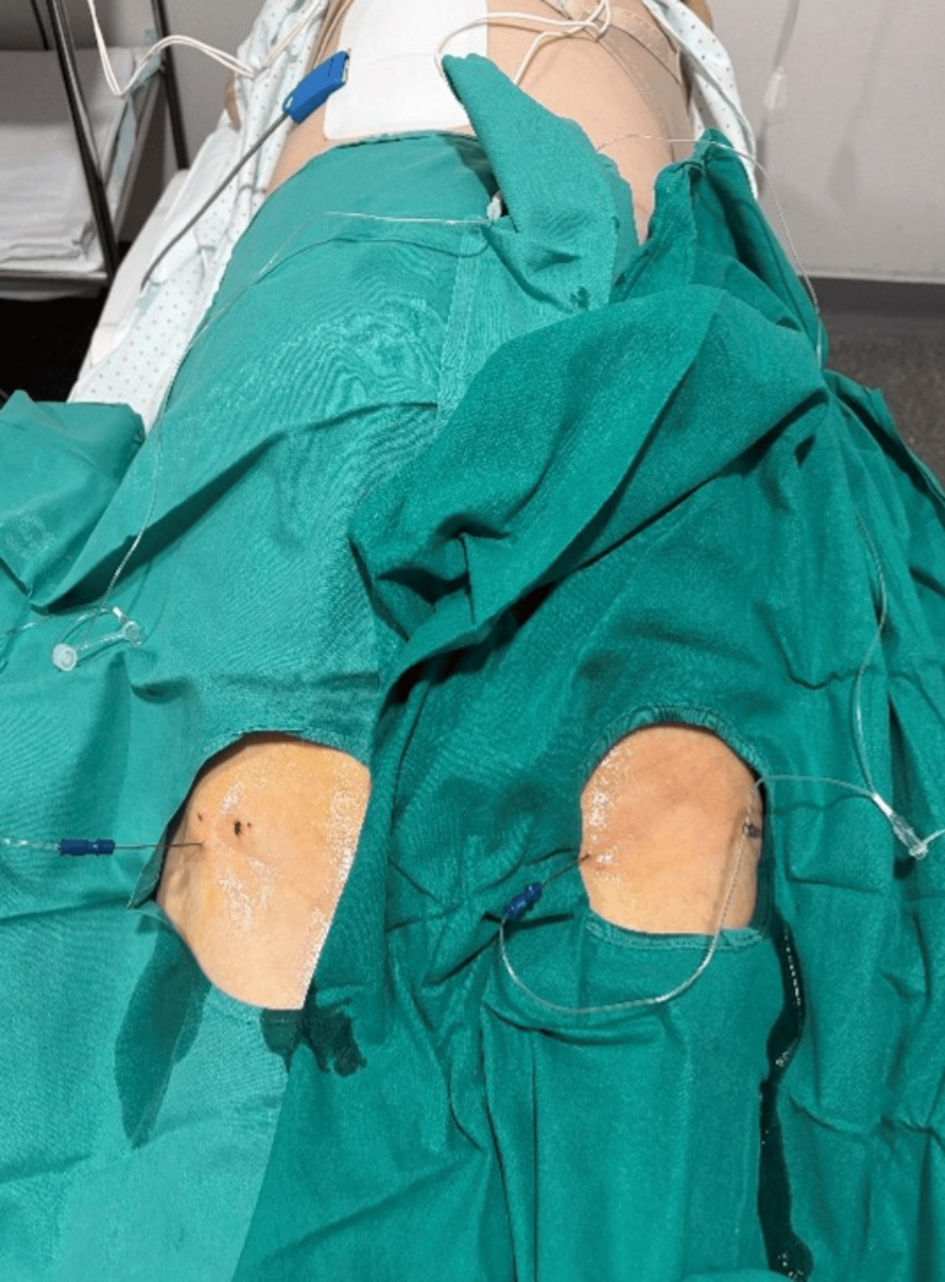
Needle disposition while applying radiofrequency.

Firstly, we applied lidocaine (2 mL) as local anesthesia. The radiofrequency needle was advanced under real-time ultrasound guidance toward the sciatic nerve. Correct needle placement was confirmed using sensory and motor stimulation according to standard protocols prior to PRF application [1,3] (Figures 2, 3).

Pulsed radiofrequency was delivered at a target temperature of 42°C, with a pulse width of 20 ms, a frequency of 2 Hz, and a total duration of 240 seconds at 45 V, consistent with parameters commonly reported in the literature for peripheral nerve neuromodulation [3,4,6].

The patient's blood pressure was monitored for half an hour. After that, the patient was discharged home with recommendations for 24 hours of rest and local application of cold compresses if he experienced any discomfort.

The following variables were collected: age, sex, diagnosis, prior analgesia (first-line analgesia: paracetamol, non-steroidal anti-inflammatory drug (NSAIDs); second-line analgesia: weak opioids (tramadol); third-line analgesia: strong opioids (morphine, fentanyl, buprenorphine, oxycodone, hydromorphone), presence of complications, and percentage of perceived overall improvement. Pain intensity was assessed using the VAS, and quality of life was assessed using the EuroQol-5D questionnaire, both before and after the procedure.

The protocol consisted of an initial medical consultation, during which the patient received an initial evaluation, baseline data were collected, and the procedure was explained. Subsequently, after being placed on our service's waiting list, the patient underwent the procedure two to four weeks later. Finally, at six months, a final evaluation was conducted, usually by telephone, during which the research data was collected. We can say that the follow-up period was six months, from the time the patient underwent the procedure until the telephone consultation.

A descriptive analysis was performed using frequencies and means ± standard deviation. To compare pre- and post-treatment changes in VAS, EuroQol-5D, and analgesia consumption, the paired Student's t-test was used (after verifying normality with the Shapiro-Wilk test). A p-value < 0.05 was considered statistically significant.

## Results

Forty-five patients were excluded, resulting in a final analysis of 266 patients. The reason the forty-five patients were excluded was that they did not attend the follow-up appointment, or because they developed a neurological deficit that prevented the correct assessment of pain. Of the total, 152 (57.14%) were women and 114 (42.86%) were men, with a mean age of 67.8 ± 13.13 years. 26 (9.78%) patients were on first-line analgesia, 120 (45.11%) on second-line analgesia, and 120 (45.11%) on third-line analgesia. The VAS and EuroQol-5D scores before the procedure were 6.52± 0.8 and 0.265 ± 0.085, respectively.

The indications for the procedure, along with their respective VAS and EuroQol-5D scores before the procedure, are presented in Table 1.

**Table 1:** VAS and EuroQol-5D before the procedure according to diagnosis VAS = Visual Analogue Scale, SD = standard deviation, CRPS = complex regional pain syndrome

Diagnosis	n	%	Previous VAS (± SD)	Previous EuroQol – 5D (± SD)
Knee pain (osteoarthritis and painful prosthesis)	112	42.11	6.38 ± 0.66	0.265 ± 0.084
Distal neuropathic pain in the lower limb (radicular pain, peripheral neuropathy and others)	85	31.95	6.51 ± 0.84	0.272 ± 0.08
Post-traumatic or osteoarthritis ankle or foot pain	26	9.77	6.69 ± 0.88	0.267 ± 0.077
Complex regional pain syndrome (CRPS) or phantom limb pain	18	6.77	7.0 ± 0.91	0.23 ± 0.103
Neurogenic claudication due to spinal canal stenosis	18	6.77	6.67 ± 1.03	0.245 ± 0.09
Vascular ischemia in the lower extremities	7	2.63	6.57 ± 0.53	0.312 ± 0.115

Following the procedure, global clinical improvement (≥ 3-point reduction on the VAS) was observed in 181 (68.05%) patients; however, only 61 (33.83%) were able to reduce their analgesic consumption, at least by one line less. The percentage of improvement according to diagnosis was 83.33% for neurogenic claudication, 80.77% for ankle/foot pain, 80% for distal neuropathic pain, 71.42% for lower limb ischemia pain, 55.55% for CRPS/phantom limb pain, and 55.37% for knee pain. Only minor complications (transient post-puncture pain and residual hematoma) occurred in 11 (4.14%) cases. The mean VAS and EuroQol-5D scores in the group of patients who improved were 2.27 ± 1.06 (-4.25, p<0.0001) and 0.596 ± 0.114 (+0.331, p<0.0001), respectively. The mean duration of improvement was 5.11 ± 1.43 months, with effects persisting for up to 10 months in some patients. The analysis of clinical improvement after the procedure was performed according to the diagnosis shown in Table 2 and Table 3, highlighting better results in the group with distal neuropathic pain in the lower limbs, CRPS, and phantom limb pain.

**Table 2:** Changes in VAS after sciatic nerve PRF after diagnosis PRF = pulsed radiofrequency, VAS = Visual Analogue Scale, SD = standard deviation, CRPS = complex regional pain syndrome

Diagnosis	n	Baseline VAS (mean ± SD)	Post procedure VAS (mean ± SD)	Paired t test	p value
Knee pain (osteoarthritis and painful prosthesis)	62	6.38 ± 0.66	2.34 ± 0.85	-4.04	p < 0.0001
Distal neuropathic pain in the lower limb (radicular pain, peripheral neuropathy and others)	68	6.38 ± - 0.66	2.13 ± 1.17	-4.38	p < 0.0001
Post – traumatic or osteoarthritis ankle or foot pain	21	6.38 ± 0.66	2.38 ± 1.20	-4.31	p < 0.0001
Complex regional pain syndrome (CRPS) or phantom limb pain	10	6.38 ± 0.66	2.30 ± 0.95	-4.70	p < 0.0001
Neurogenic claudication due to spinal canal stenosis	15	6.38 ± 0.66	2.47 ± 1.13	-4.20	p < 0.0001
Vascular ischemia in the lower extremities	5	6.38 ± 0.66	2.00 ± 1.87	-4.57	p < 0.0008

**Table 3:** Changes in EuroQol-5D after sciatic nerve PRF after diagnosis PRF = pulsed radiofrequency, VAS = Visual Analogue Scale, SD = standard deviation, CRPS = complex regional pain syndrome

Diagnosis	n	Baseline EuroQol-5D (mean ± SD)	Post procedure EuroQol-5D (mean ± SD)	Paired t test	p value
Knee pain (osteoarthritis and painful prosthesis)	62	0.265 ± 0.084	0.579 ± 0.126	+0.314	p < 0.0001
Distal neuropathic pain in the lower limb (radicular pain, peripheral neuropathy and others)	68	0.272 ± 0.080	0.620 ± 0.110	+0.348	p < 0.0001
Post – traumatic or osteoarthritis ankle or foot pain	21	0.267 ± 0.077	0.579 ± 0.100	+0.312	p < 0.0001
Complex regional pain syndrome (CRPS) or phantom limb pain	10	0.230 ± 0.103	0.585 ± 0.096	+0.355	p < 0.0001
Neurogenic claudication due to spinal canal stenosis	15	0.245 ± 0.090	0.572 ± 0.080	+0.327	p < 0.0001
Vascular ischemia in the lower extremities	5	0.312 ± 0.115	0.653 ± 0.195	+0.341	p < 0.009

## Discussion

In response to the rising prevalence of chronic pain, new strategies are being developed to address the diverse conditions affecting patients and, ultimately, to improve their quality of life. Prolonged use of analgesics is associated with relevant adverse effects, including gastrointestinal, renal, and cardiovascular toxicity with NSAIDs, as well as sedation, constipation, tolerance, and dependence with opioids. Adherence is another major concern, as complex regimens and poor tolerability often lead to suboptimal compliance and reduced effectiveness. For this reason, the use of pulsed radiofrequency for the treatment of nociceptive and neuropathic pain has been growing in recent years [6,16]. However, there are only a few studies currently published on ultrasound-guided pulsed radiofrequency of the sciatic nerve, its clinical applicability, and safety.

In this study, we describe our experience with this technique for the treatment of lower-limb pain (radicular, peripheral neuropathic, knee, ankle/foot, CRPS/phantom limb, neurogenic claudication, and distal vascular ischemia) in a cohort of 266 patients, observing a global improvement in 181 (68.05%) cases, with a mean duration of 5.11 ± 1.43 months. In addition, we observed a very low prevalence of mild, transient adverse effects: only in 11 cases (4.14%). It is noteworthy that the marked reduction in VAS and EuroQoL-5D scores contrasts with the relatively modest decrease in analgesic consumption. This discrepancy may be explained by the multifactorial nature of chronic pain management, as improvements in pain perception, functional capacity, and quality of life do not necessarily translate into proportional reductions in medication use, particularly in patients with long-standing pain, established prescribing patterns, or concomitant comorbidities requiring continued pharmacological treatment [16].

Experimental studies in animal models show that sciatic nerve PRF reduces the expression of calcitonin gene-related peptide (CGRP) in the dorsal root ganglion (DRG), which correlates with improvements in neuropathic pain signs (hyperalgesia and allodynia) [4]. These findings could suggest that PRF has a neuromodulatory effect that alters pain transmission, which would support our results, especially in patients with distal neuropathic pain, neurogenic claudication, and CRPS/phantom limb pain groups in which we observed the highest rates of improvement.

Although there are meta-analyses and systematic reviews concluding that the evidence on the use of PRF for neuropathic pain is promising, their results are limited by small sample sizes and/or data heterogeneity [17]. On the other hand, there are few randomized trials on peripheral nerve PRF for neuropathic pain, including one double-blind trial whose results showed no superiority of PRF over placebo in post-traumatic peripheral neuropathic pain [18]. With regard to specific treatment of the sciatic nerve, there are very few case reports or case series describing the use of sciatic nerve PRF for refractory pain in certain clinical situations, with good results [19]. A recent retrospective study in Switzerland observed an overall improvement in 12 out of 17 patients (71%) with distal neuropathic pain of approximately three months duration, while 10 (67%) were able to reduce or discontinue their analgesic medication during that time [16].

Regarding its utility in the treatment of chronic knee pain, a prospective study in 25 patients (47 procedures) showed a significant reduction in VAS scores over three months, with no procedure-related complications, highlighting the safety and feasibility of the ultrasound-guided procedure [19]. Another recent study on the utility of ultrasound-guided pulsed radiofrequency for the treatment of chronic knee pain observed a significant clinical improvement in VAS, EuroQol-5D, and the WOMAC Index for at least four months after pulsed radiofrequency of the sciatic nerve [20].

With respect to neuropathic, post-traumatic, or degenerative ankle and foot pain, there are case series on pulsed radiofrequency (PRF) of the sciatic nerve branches (peroneal, tibial, or sural) demonstrating effectiveness in treating post-traumatic pain, pain from plantar fasciitis and metatarsalgia [21-23], and distal neuropathic and nociceptive pain in the ankle and foot [24,25].

Concerning phantom limb pain, there are only isolated case reports on its effectiveness, with significant clinical improvement and pain relief lasting up to four to six months [26,27]. Likewise, the utility of sciatic nerve PRF has been described in case series of CRPS and post-traumatic peripheral neuropathy, where a significant improvement in the VAS score was observed for six to eight months [9,10].

Our data, comprising 266 patients, constitute a large real-world registry evaluating the use of this technique, providing additional clinical evidence on its feasibility and utility in current clinical practice. The fact that the highest improvement rates were observed in subgroups with predominantly neuropathic or neurogenic pain (distal neuropathic pain, neurogenic claudication, CRPS/phantom limb) supports the hypothesis that PRF has a neuromodulator effect, rather than merely a transient analgesic effect [12].

Limitations

As a retrospective, observational study without a control group, it is not possible to rule out a placebo effect, spontaneous regression, or the contribution of other concomitant therapies, so these results should be interpreted with caution until controlled, ideally randomized studies of this procedure are available, as we cannot assume causality. An additional limitation of this study is the potential treatment bias related to concomitant pain management therapies during the follow-up period. Although a reduction in analgesic consumption was observed after PRF, changes in medication dosage, treatment adjustments, or adjunctive interventions (by the primary care physician) during the follow-up period may have acted as confounding factors influencing clinical outcomes. Therefore, future studies should prospectively standardize and closely monitor all pain control treatments administered throughout the PRF treatment course. Furthermore, the study does not record the duration of pain prior to the procedure, which would have given greater rigor to the results.

## Conclusions

Ultrasound-guided pulsed radiofrequency of the sciatic nerve may represent a minimally invasive, safe, and potentially effective therapeutic option for nociceptive, neuropathic, or mixed lower-limb pain. These findings are consistent with previous reports on peripheral nerves, providing additional real-world evidence and supporting its use in refractory chronic pain. Nevertheless, the current evidence remains limited, highlighting the need for well-designed studies to establish its definitive role in pain management.
